# Supplementary material for: Urban metagenomics uncover antibiotic resistance reservoirs in coastal beach and sewage waters
Source: Microbiome. 2019 Feb 28;7:35. doi: 10.1186/s40168-019-0648-z (PMC6396544; doi:10.1186/s40168-019-0648-z)
Supplement: Supplementary file 1 — Supplementary figures. (PDF 426 kb) [file 40168_2019_648_MOESM1_ESM.pdf]

SUPPLEMENTARY INFORMATION

City-wide metagenomics uncover antibiotic resistance reservoirs in urban beach and sewage waters

Fresia *et al.*

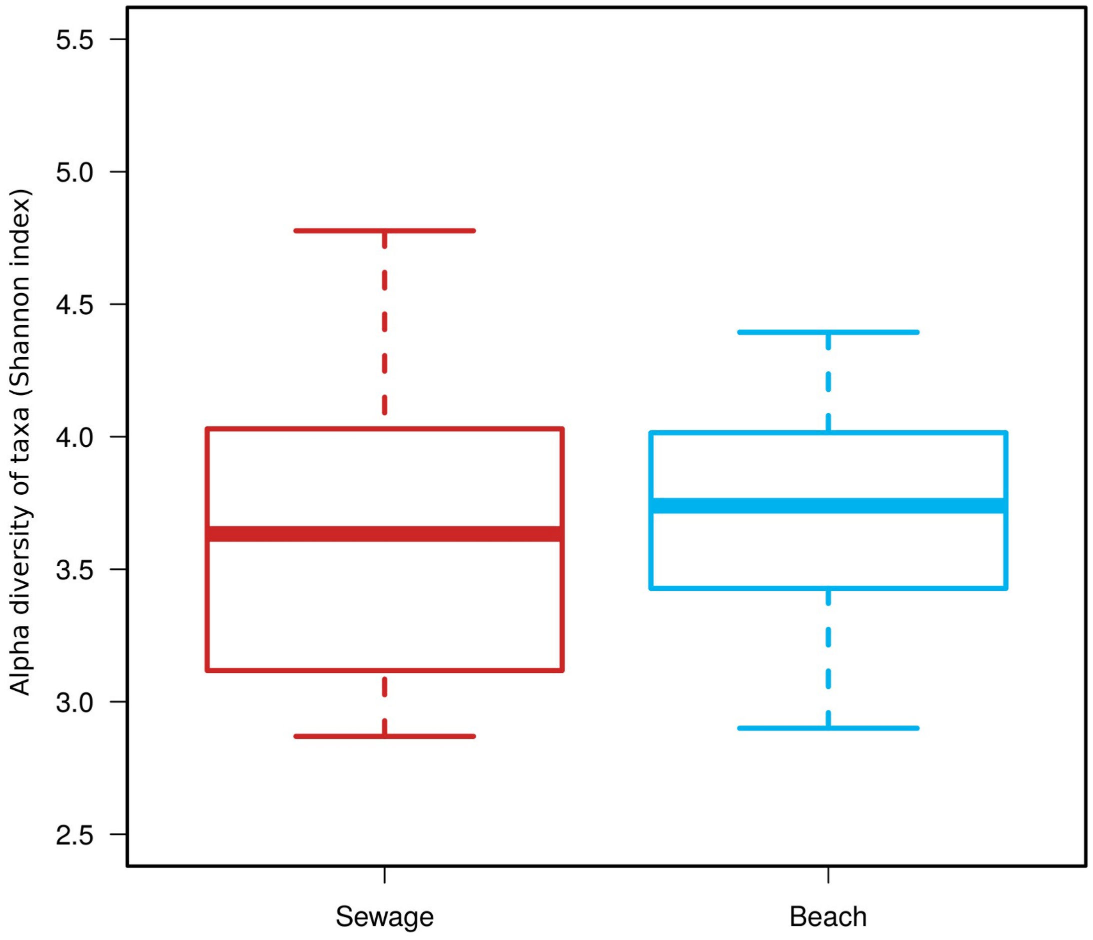

Supplementary Figure 1. Alpha diversity plots. Boxplots show the Alpha diversity according to the Shannon index calculated for sewage (red) and beach (blue) samples.

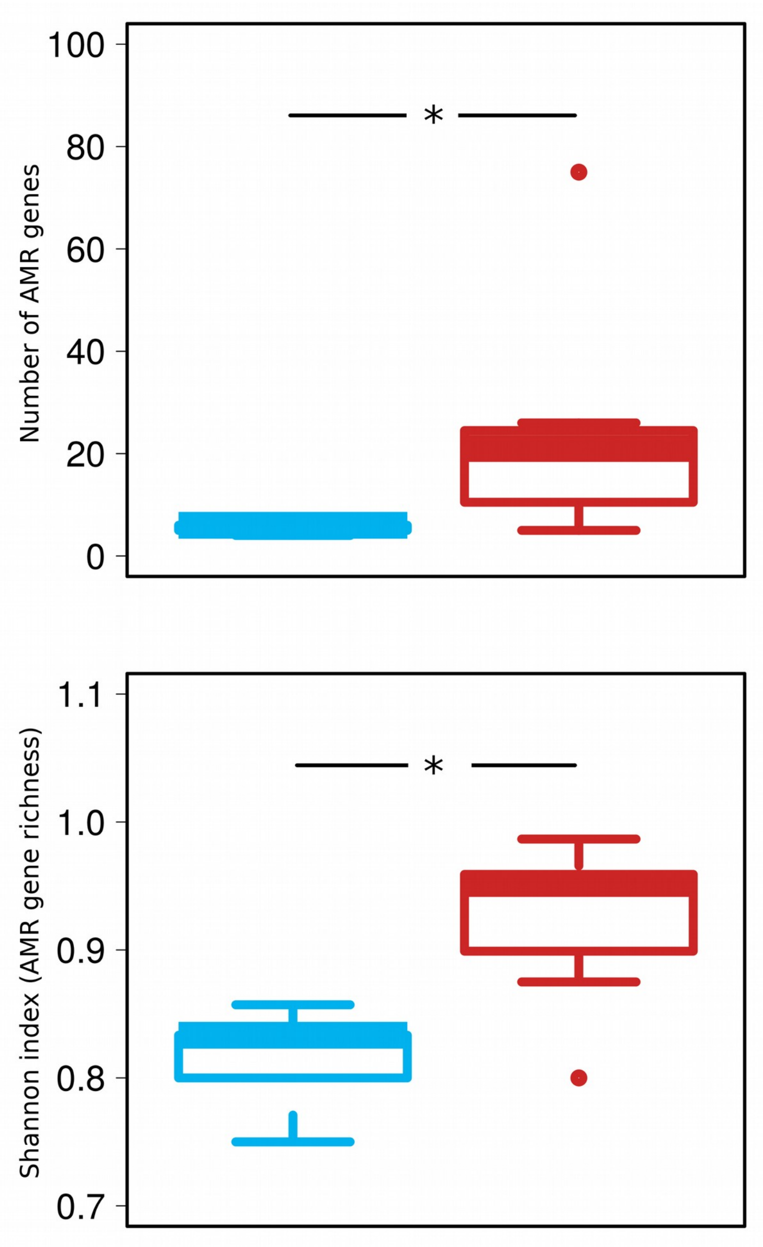

Supplementary Figure 2. AMR gene count and richness. Boxplots show the number of AMR genes recovered from beach (blue) and sewage (red) samples, and the AMR gene richness in beach (blue) and sewage (red) samples. The asterisk indicate $p < 0.01$ (Mann-Whitney U test).

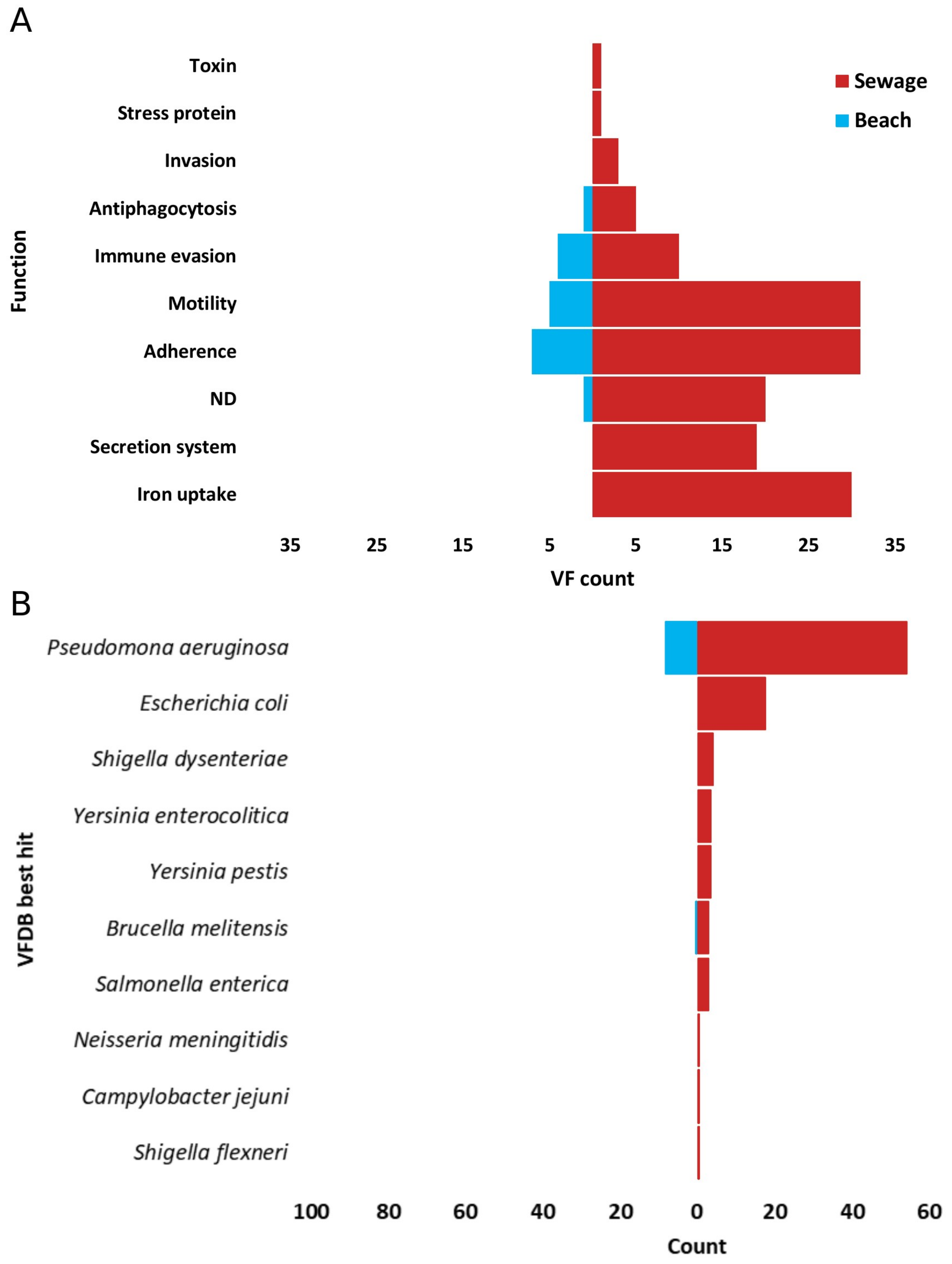

Supplementary Figure 3. Distribution of virulence factor genes. A) Barplots show virulence gene counts in sewage (red) or beach (blue) samples, classified by virulence function. B) Barplots show virulence gene counts in sewage (red) or beach (blue) samples, classified by the top-hit species occurying in the database.
